# Suits for Malpractice

**Published:** 1858-03

**Authors:** 


### Editors' Table.

Suits for Malpractice.—In what light are we to regard the growing fondness for bringing suits for malpractice, alleged or real, against medical men, and, more particularly, against those who take charge of surgical cases? Shall we call the course thus pursued prosecution or persecution,—an honest claim for reparation on the part of the patient for injury causelessly and ignorantly inflicted on him, or a nefarious scheme for his not only evading the payment of fees for services rendered, but also for extorting money from his professional attendant, on the plea that these services were not sufficiently full and complete, and have not been attended with the success anticipated? The stupidity, obstinacy, and even drunkenness of the patient, by which professional injunctions are misunderstood, opposed, or neglected, do not seem to be always a bar in the minds of a jury against mulcting the surgeon in a considerable sum, in the shape of damages. Other obstacles to the hoped-for cure, depending on constitutional feebleness and unsoundness, either inherent, or the result of intemperance and a generally dissolute life, are ignored or determinedly overlooked, in the intentness of the maimed or recently deformed man to extort money from the purse of his doctor, in place of dropping a handsome honorarium into this seldom well-replenished receptacle. Under circumstances like these, and some others which might be adduced, an action for malpractice goes far beyond the ingenuity which suggests a cross suit: it approaches rather, but in a more formal manner, and with a show of legality, to the conduct of a beggar, who, while in the act of receiving a modicum to relieve his immediate necessities, unexpectedly presents a pistol to the breast of his benefactor, and insists on the latter handing over to him his purse with all its contents. We may expect, if juries are so ready to encourage by their verdicts this new phase of extortion, to find a hitherto unlooked-for addition to the beneficial societies which pay their sick members a certain sum weekly. He who is laid up with a fractured limb or a dangerous wound, or with a disorder for the relief or cure of which an operation has been performed, will receive his stated allowance from the society during the time of his confinement to the house; and, when he gets about, if he does not walk as straight, or move his arm, or have had his sight restored, as he expected, he may, with the assistance of a good special-pleader, and a willing jury, procure a round sum from his attending surgeon, by accusing the latter of malpractice.

Physicians labor under the disadvantage of being regarded as constituting a priviliged class, at the same time that they are actually denied the possession of any substantial privilege. They enjoy no exclusive honors or emoluments, nor exemption from taxes, public or private, direct or incidental: but they get credit for having a charmed life, through which they are supposed to enjoy immunity from disease, and even from feelings of fatigue and exhaustion incident to exposures of all kinds, and to the calls made on them at all hours and seasons. When pestilence stalks abroad, and prostrates its victims in all directions, they are honored with peculiar deference and protestations of confidence and regard,—they again become a privileged class: they have the undisputed privilege of being courageous in the midst of danger, and of submitting to a wear and tear of mind and body beyond what would seem to be the limits of human endurance. If some of them sink under the accumulated load, and perish, victims to their heroic devotion in the cause of humanity, some words of regret are uttered in the immediate circle of their patients, a few lines of eulogy find their way to a newspaper; but the community, in whose behalf they so nobly battled, never asks about, never hints at making any provision for their families, left, perhaps, in straitened circumstances, if not in destitution. And the survivors from the pestilence—what is their reward? A vote of thanks, and, perhaps, a silver pitcher, with their remembrance of the thousands on whom, at all hazards to their own lives, they have lavished their time and their services, without, except in a comparatively small number of instances, any pecuniary return. There is yet another privilege conceded to physicians—viz.: to live without income, and in noble disregard of fees. For one instance of a gracefully tendered addition to the amount of their bills by a restored patient, there will be found a hundred of begrudged or deferred payment; and a manifest desire, under one pretext or another, to cut down the amount. The gratuitous services rendered by physicians to the poor, and the nominal fees from many in reduced circumstances, so far from exciting responsive feelings among the rich, would seem, in some instances, to prompt the latter to additional vigilance in scrutinizing their medical accounts, for fear that they might by chance contribute indirectly to this fund of benevolence, by paying what they are sometimes pleased to call high charges.

In these remarks we do not speak of the motives and feelings which prompt medical men to discharge their high duties; but only of the slim appreciation of their services by the community, and a readiness to tax them in every possible manner. We are disposed to regard as an extension of this jealous fear of remunerating a physician for his services to the sick, the growing desire to seize on any plausible pretext for levying fines on him for alleged malpractice. The community is not entitled to assume the tone of a liberal and discriminating benefactor, and to say to physicians, "You are well and regularly paid in every case in which you perform your duty; and it is but fair that you should suffer by fine when you are wanting in its discharge." The first part of the proposition is not true, and the second, intended to be its sequence, falls to the ground.

Looking at the subject from a higher stand-point, shall we be told, that the State, ever watchful of the welfare of its citizens, guards their health with peculiar care, and not only prohibits any tampering with it by ignorant pretenders and quacks, but provides the means for educating a body of competent physicians, whose knowledge of and familiarity with public and private hygiene and the cure of diseases, prepare them for every emergency. Consistently with ihis intention, even if provision be not made, every encouragement is of course given for prosecuting the study of anatomy, so indispensable to the obtaining a knowledge of the structure of the human body,—a knowledge which lies at the foundation of all surgery, and without which the treatment of fractures, dislocations, and wounds is necessarily uncertain and hazardous. But what must be the surprise and indignation of the benevolent and philosophical inquirer to learn that, so far from this bright picture being the true one, dissection of the dead, and the procuring a subject for the purpose, are regarded in the light of *magnum delictum*, which subjects the unfortunate seeker after knowledge to fine and imprisonment, as if he had committed felony or a breach of the peace by a violent assault on his neighbor. Still, although prohibited by these penalties from learning surgery in a fitting manner, the practitioner is supposed to have acquired, by some intuitive process or species of magic, a knowledge sufficient to guide him through all difficulties. Hence it may happen, that in the very same community, and by the very same men, acting as jurors, a student of medicine will be punished for an attempt to acquire a knowledge of anatomy, and, in a few years from that time, he will be punished, in the shape of damages, for betraying ignorance, in his treatment of a fracture or a dislocation of parts of the structure and mechanism which belong to anatomy.

Not only does legislation fail to make suitable endowments for medical education, but it draws no distinction, either in the way of warning or encouragement, between those who are educated and prepared to take charge of the sick, and those who are ignorant and illiterate, and yet engage in the same mission. Time shows the difference between the two, but not until heavy penalties are paid in preventible deaths, and in lasting injury to the health of a large number of persons. After all, the public is more prone to receive with favor bold and shameless pretenders, than to inquire into the merits of unobtrusive worth; and it inclines a more willing ear, and affords more direct patronage, to a natural bone-setter than to the most experienced surgeon. The fellow-feeling will go so far as to make it deal more leniently with the former after his mischievous manipulations, than with the latter under the accusation, even without adequate proof, of inattention or mistake in his practice in a particular case charged.

Having shown that no claim in equity can be made by the community, or even by the State itself, for the punishment of malpractice on the part of a medical man, we have next to inquire into the real position of the latter, his actual responsibilities, and the tribunal to which he is properly amenable. This part of the argument will engage our attention in a future editorial talk.

